# Late* Ps. aeruginosa *Inguinal Mesh Infection 12 Years after the Initial Operation: Report of the Case and Short Review of the Literature

**DOI:** 10.1155/2017/4385913

**Published:** 2017-09-27

**Authors:** Dimitrios Filippou

**Affiliations:** ^1^Department of Laparoscopic Surgery and Surgical Oncology, Neo Athinaion Hospital, Athens, Greece; ^2^Department of Anatomy and Surgical Anatomy, Medical School, University of Athens, Athens, Greece

## Abstract

Inguinal hernia mesh repair is one of the most frequent operations performed worldwide. The Lichtenstein technique and its various modifications are the most popular operations for groin hernia repair. The rate of surgical site infection (SSI) following inguinal hernia repair ranges between 0 and 14% in various series. Most of these infections developed early postoperatively. The incidence of late mesh infection following open inguinal hernia repair still remains unclear and highly variable. Late deep mesh infections are relatively rare specially after more than 10 years. The most common pathogens reported in the literature are* E. coli* and* St. aureus*. The infection is treated by conservative means initially but in case of failure then the mesh should be removed surgically. A unique case of a patient with very late (chronic) mesh infection is presented. The infection was due to* Pseudomonas aeruginosa*, which occurred 14 years after the initial operation and presented as subcutaneous fistula.

## 1. Introduction

Inguinal hernia mesh repair is one of the most frequent operations performed worldwide. The Lichtenstein technique and the various modifications of the method are nowadays the most popular operations for inguinal hernia repair and the tension-free mesh repair is considered the golden standard mainly due to reduced rates of recurrence and technical ease of the operation [1, 2].

Lichtenstein technique is an open surgical technique for inguinal hernia repair based in the enhancement of the weakened inguinal floor by tension-free placing of a polypropylene mesh [3, 4].

Over the past years there has been a continuous debate concerning the possible and the type of prophylactic preoperative administration of antibiotics. Routine antibiotic prophylaxis is still controversial even in procedures where a synthetic implant is used like Lichtenstein technique [5, 6]. Antibiotic prophylaxis and mesh-saving operations are suggested by several authors but are not sufficient to eradicate the infection in most of the cases [7, 8]. The rate of surgical site infection (SSI) following inguinal hernia repair ranges between 0 and 14% in various series [5]. Most of these infections developed early postoperatively. The incidence of late mesh infection following open inguinal hernia repair still remains unclear and highly variable. Late mesh infection in inguinal hernia might be related to several factors including the surgical technique, the type of mesh, and the prophylactic chemotherapy and varies significantly between various series [9]. In Lichtenstein series the overall infection rate was 0.003%, while in another series report by Shulman et al. the reported infection rate was 0,8% [10, 11].

The most common bacteria in chronic mesh infections following open inguinal hernia repair are* Staphylococcus aureus, Escherichia coli*, Enterococci, and coagulase-negative* Staphylococcus* [7].

A unique case of a patient with very late (chronic) mesh infection is presented. The infection was due to* Pseudomonas aeruginosa*, which occurred 14 years after the initial operation and presented as subcutaneous fistula.

## 2. Report of the Case

**Figure 1:**
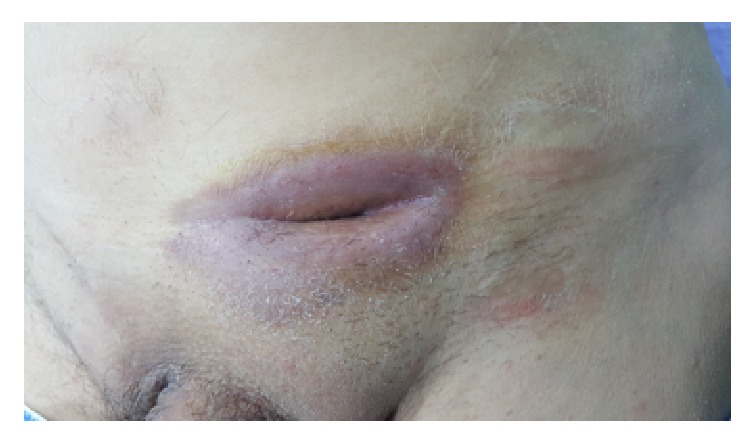
Photo of the fistula opening. The fistula remained after deep mesh infection with* Ps. aeruginosa *despite the initial conservative treatment and surgical intervention.

A 62-year-old male proceeded to our Hospital complaining of a mild edema, redness, and pain in the left groin area. The clinical examination revealed a small fistula in the affected area. The patient was receiving medication for epilepsy since the age of seventeen (tabl. Valproic acid 500 mg ×3). He had received appendectomy (1969), right inguinal hernia repair with mesh (1990), left inguinal hernia repair with mesh (2004), endoscopic colon polyps resection (2014 and 2015), and turis prostatectomy (2014). The blood exams revealed mild leukocytosis (WBC: 12,000/*μ*L) and slightly elevated CRP (3.2 *μ*g/L). The patient had visited a doctor on external basis two days ago who prescribed him cefuroxime 500 mg ×2. No other medical problems or other comorbidity factors were referred. The culture of the fluid received from the fistula opening revealed infection from* Pseudomonas aeruginosa*, sensitive to ciprofloxacin. We decided to treat the patient conservatively by administrating ciprofloxacin (500 mg per os twice a day) and clean the trauma with hydrogen superoxide (H_2_O_2_) via the fistula opening (twice a day). Ten days later we reexamined the trauma fluid and although no microorganism was identified in the fluid cultures the fistula still remained (Figure 1). During this period there was not any progress in the fistula closing so it was decided that the patient should be treated surgically. The patient submitted to surgical fistulectomy (complete removal of the fistula tract in health margins) under local anesthesia, after marking the fistula tract with blue de methylene. The fistula tract related closely to the mesh but there was not any evidence of direct contact. The decision for keeping the mesh was based mainly on the fact that the mesh did not get colored by blue dye, which supported the hypothesis that the mesh may have not been infected. The postoperative course of the patient was uncomplicated but 3 months after the operation the fistula recurred.

**Figure 2:**
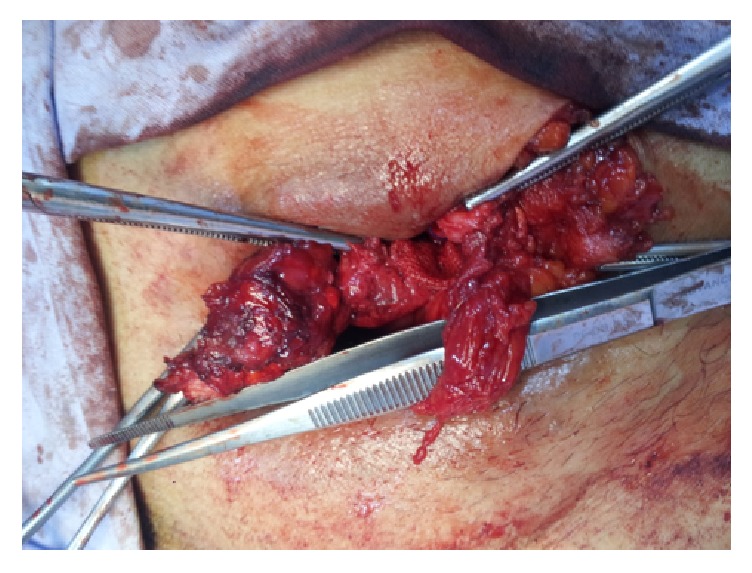
The vas deferens and the vessels where recognized in full length and preserved, while the mesh was totally removed.

**Figure 3:**
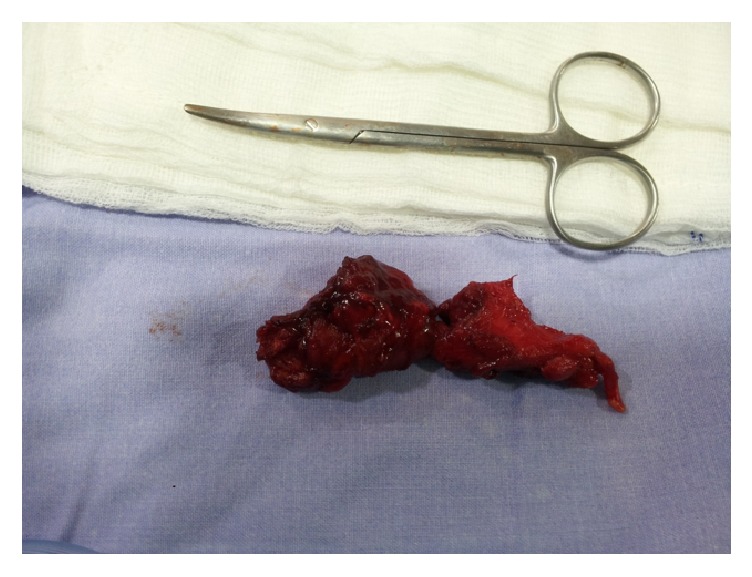
The mesh was fully removed. No sutures were used and the trauma closed only with nylon sutures. The internal scar was kept where it was possible to avoid possible hernia recurrence in the future.

We decided to reoperate on the patient and remove the mesh. The operation took place with the patient in supine position under general anesthesia. The vas deferens and the vessels were recognized in full length and preserved, while the mesh was totally removed (Figures 2 and 3). A Penrose drain was placed and the skin was closed with three sutures. No other sutures were placed to avoid possible contamination. The postoperative course was uncomplicated and the patient was discharged the next day. The drain was removed 2 days later. Nine months after the operation there are no signs of the fistula or the hernia recurrence.

## 3. Discussion

Lichtenstein technique and its various modifications for tension-free inguinal hernia repair have gained recognition and adoption among the surgeons worldwide. Nowadays, most of the surgeons prefer to use one or more of these techniques to achieve a painless and effective inguinal hernia repair. The development of new materials and improved meshes resulted in diminishing morbidity and recurrence rates [9]. However the late-onset deep mesh infection still remains an unpredictable complication. A detailed search of the literature concerning late-onset deep mesh prosthesis was performed but no other similar cases were found. The latest mesh infection we managed to find was due to* St. aureus* infection 17 years after the initial operation, while the latest infections with* Ps. aeruginosa *was 20 months after the mesh placement [10–13]. The most common microorganism identified in late-onset deep mesh infections in inguinal hernias according to the existing data is* St. aureus*, while* Ps. aeruginosa *is considered as a rare pathogen. In a very interesting report the incidence of late-onset deep mesh infection following hernia repair studied in a series of 2666 consecutive patients. The authors described 8 cases of late-onset deep mesh infection. Only two of these late deep infections complicated inguinal hernia repairs. The incidence was 0.24%, while in another large series it increased up to 0.34% [12, 13]. The published work of Delikoukos et al. is of great importance because it was scheduled to study and report the experience of late deep mesh infection following inguinal hernia repair. The authors concluded that the true incidence is yet to be established [12].

Various risk factors that may be associated with late-onset deep mesh infection have been proposed but there is lack of evidence. Most authors agree that aseptic technique, prior superficial wound infection, and perioperative chemoprophylaxis may have a potentially critical role [2, 7, 12]. Some authors tried to evaluate the possible role of the type of the mesh used or the fixation material but the results seem to be insufficient and confusing [7, 11, 13]. Delikoukos et al. suggested that late graft infection does not seem to correlate with perioperative chemoprophylaxis administration or the presence of previous superficial wound infection. Furthermore, graft infection does not seem to correlate with neither the type of mesh inserted nor the fixation material. On the other hand the authors note that the use of synthetic materials seems to increase the overall incidence of late mesh infection [12]. More and better organized studies with larger number of patients are required to clarify the possible role of the synthetic material used in meshes and for fixation in late deep infections [14]. Although several authors suggest that the number of the present bacteria, the bacterial virulence, and the wound microenvironment may have a critical role in the development of late-onset deep mesh infection there are not enough data to support this theory. It is more possible that these factors may play a more crucial role in the early infections than the late ones especially in those that develop several years after the operation [4–6, 13].

The diagnosis of infection can be easily set by clinical examination because of the symptoms that are typical. Differential diagnosis is required in most of the cases to exclude other possible causes as skin infections and superficial wound infections [9]. In most of the cases imaging techniques may be used although there is no evidence that they can accurately predict a possible mesh infection. In rare cases such as in our presented case fistula may be developed and help us to establish a correct diagnosis. The therapeutic algorithm includes conservative and surgical procedures [13, 15].

Several authors suggest that abscess puncturing and antibiotic therapy should be considered as first choice and may provide cure mesh removal. Other interventions for conservative treatment of deep mesh infections, like wound vacuum assisted devices, have also been used and reported by various authors but the results are not encouraging [7]. If the initial conservative treatment fails then surgical removal of the mesh may be necessary [15]. A general therapeutic algorithm suggests that conservative treatment for deep mesh infection after inguinal hernia repair should be attempted initially but not for more than 2 weeks. If the conservative means fail to treat the infection then surgical removal of the mesh should be attempted [12–15]. Many authors tried to answer the difficult question if the infected mesh can be saved [9, 10]. Till now there is not a clear answer. Many identified factors that are associated may play a critical role. The lack of established guidelines raises the interest for further clinical research. In the present case the lack of data concerning the initial operation, the technique used, the type of the mesh, and the possible perioperative complications confused the treating strategy [2, 10]. In the present case the selection of the treating algorithm is based on clinical criteria. The problem is treated initially by conservative means as most authors suggest. The lack of clinical improvement (no progress in fistula closing) within the first ten days suggested that surgical intervention was required [2, 10, 13]. The inability to prove intraoperatively contact between the fistula tract and the mesh suggested that the mesh may be salvaged and that fistulectomy may be suitable for this patient. The present case not only is unique but also presents a great educational interest for similar complicated cases.

Late deep mesh infections following inguinal hernia repair are rare although their incidence seems to increase following the increasing use of synthetic materials. The exact mechanism and the related factors have not been identified and large studies are required to clarify them.

## Data Access

Data are available on request from Dimitrios K. Filippou, M.D., Ph.D. (d_filippou@hotmail.com).
